# Salubrity of Philadelphia

**Published:** 1848-04

**Authors:** 


### SALUBRITY OF PHILADELPHIA.

The able editors of the "Western Journal of Medicine and Surgery," infer from the fact that but two medical students, in a class of 406, have died there during the last session, that Louisville is signally healthy. What then must be the healthfulness of Philadelphia, when only two had died up to the 1st of March last in about 1200. The Catalogues of the University of Pennsylvania and the Jefferson Medical College have alone been published. In these two schools of the *five*, there were nearly one thousand students.

